# Activation of α-7 Nicotinic Acetylcholine Receptor Reduces Ischemic Stroke Injury through Reduction of Pro-Inflammatory Macrophages and Oxidative Stress

**DOI:** 10.1371/journal.pone.0105711

**Published:** 2014-08-26

**Authors:** Zhenying Han, Fanxia Shen, Yue He, Vincent Degos, Marine Camus, Mervyn Maze, William L. Young, Hua Su

**Affiliations:** 1 Center for Cerebrovascular Research, Department of Anesthesia and Perioperative Care, University of California San Francisco, San Francisco, California, United States of America; 2 Tianjin Medical University General Hospital, Tianjin, China; 3 Hôpital Pitié Salpetrière, Assistance Publique-Hopitaux de Paris (APHP), Université Pierre et Marie Curie-Paris VI and UMR INSERM 1141, Paris, France; 4 Department of Neurological Surgery, University of California San Francisco, San Francisco, California, United States of America; 5 Department of Neurology, University of California San Francisco, San Francisco, California, United States of America; University of Naples Federico II, Italy

## Abstract

Activation of α-7 nicotinic acetylcholine receptor (α-7 nAchR) has a neuro-protective effect on ischemic and hemorrhagic stroke. However, the underlying mechanism is not completely understood. We hypothesized that α-7 nAchR agonist protects brain injury after ischemic stroke through reduction of pro-inflammatory macrophages (M1) and oxidative stress. C57BL/6 mice were treated with PHA568487 (PHA, α-7 nAchR agonist), methyllycaconitine (MLA, nAchR antagonist), or saline immediately and 24 hours after permanent occlusion of the distal middle cerebral artery (pMCAO). Behavior test, lesion volume, CD68^+^, M1 (CD11b^+^/Iba1^+^) and M2 (CD206/Iba1^+^) microglia/macrophages, and phosphorylated p65 component of NF-kB in microglia/macrophages were quantified using histological stained sections. The expression of M1 and M2 marker genes, anti-oxidant genes and nicotinamide adenine dinucleotide phosphate (NADPH) oxidase were quantified using real-time RT-PCR. Compared to the saline-treated mice, PHA mice had fewer behavior deficits 3 and 7 days after pMCAO, and smaller lesion volume, fewer CD68^+^ and M1 macrophages, and more M2 macrophages 3 and 14 days after pMCAO, whereas MLA's effects were mostly the opposite in several analyses. PHA increased anti-oxidant genes and NADPH oxidase expression associated with decreased phosphorylation of NF-kB p65 in microglia/macrophages. Thus, reduction of inflammatory response and oxidative stress play roles in α-7 nAchR neuro-protective effect.

## Introduction

Stroke is the fourth leading cause of death in the United States [1]. In response to ischemic brain injury, the resident microglia and systemic macrophages are rapidly mobilized to the injury site and initiate inflammatory response [2]. Studies have reported biphasic inflammatory response after stroke [3]. Inflammation after cerebral ischemia amplifies the initial injury by linking acute responses in glia and cytokines to a secondary infiltration of immune cells into the brain. Prolonged inflammation may offer a longer window of opportunity to block secondary events that expand brain infarction and injury [3]. Inflammation at the acute stage of stroke has an adverse effect on stroke recovery [3]–[5], and modulating inflammation has been shown to promote the healing process and functional recovery [6]–[8].

Microglia/macrophages can polarize into two extreme phenotypes: pro-inflammatory (M1) and anti-inflammatory (M2), producing pro- or anti-inflammatory cytokines and chemokines, respectively [9]–[12]. Hu et al showed in a transient ischemic stroke mouse model that microglia/macrophages respond dynamically to ischemic injury, experiencing an early “healthy” M2 phenotype, followed by a transition to a “sick” M1 phenotype [12]. Thus, reduction of the M1/M2 microglia/macrophage ratio both at the acute and chronic stages of stroke might improve stroke recovery.

Reducing inflammation at the acute stage of ischemic stroke has been shown to attenuate brain damage and improve functional outcomes [8]. The physiological regulation of the innate immune system has been used in the treatment of infectious or inflammatory diseases [13], [14]. One of the regulatory targets is α-7 nicotinic acetylcholine receptor (α-7 nAchR), which is expressed on the surface of systemic macrophages as well as on neurons, microglia, and endothelial cells of the mammalian brain [15]–[19]. Activation of α-7 nAchR attenuates macrophage production of inflammatory cytokines and inhibits the inflammation process [13], [20], [21]. α-7 nAchR agonist treatment has reduced brain injury in a subarachnoid hemorrhage rat model [16] and conferred neuro-protection in an intracerebral hemorrhage mouse model [19]. It has also reduced neuro-inflammation and cognitive decline in mice that have been subjected to aseptic bone fracture surgery [21]. However, the mechanism of α-7 nAchR neuro-protective effect is not fully understood.

It has been reported that oxidative stress is implicated in the pathogenesis of brain injury during ischemic or hemorrhagic stroke. Nicotinamide adenine dinucleotide phosphate (NADPH) oxidase-mediated oxidative stress is recognized as one of the main mechanisms triggering pathogenic action in ischemic stroke [22], [23]. Chen et al demonstrated that mice deficient in NOX2 (one of the subunits of NADPH oxidase) decreased levels of pro-inflammatory mediators after ischemic stroke [24], associated with the activity of the inflammatory transcription factor, nuclear factor kappa B (NF-kB) [25]. It is still unclear whether activation of α-7 nAchR can modulate oxidative stress after pMCAO.

We tested the hypothesis that α-7 nAchR agonist protects brain against ischemic injury through reduction of pro-inflammatory macrophages (M1) and oxidative stress in a mouse ischemic stroke model. The mouse is used because there is no computer simulation or in vitro system can fully mimic injury response and our understanding of mouse genetics, the availability of molecular probes and antibodies for outcome analyses. The primary analysis is the behavior tests. The secondary analysis includes quantification of M1 and M2 microglia and macrophages, and oxidative stress.

## Materials and Methods

### Ethics statement

Animal experimental procedures were approved by the Institutional Animal Care and Use Committee (IACUC) of the University of California, San Francisco (UCSF), and conformed to NIH Guidelines. Animal husbandry was made available by the staff of the Animal Core Facility, and by the staff of the IACUC of UCSF, under the guidance of supervisors who are certified Animal Technologists. Veterinary care was provided by IACUC faculty members and veterinary residents located on the San Francisco General Hospital campus.

### Animals

Adult wild-type male mice (C57BL/6J, 8–10 weeks of age) were purchased from Jackson Laboratory (Bar Harbor, ME). Mice were fed standard rodent food and water ad libitum, and were housed (5 per cage) in 421×316 cm^2^ sawdust-lined cages in an air-conditioned environment with 12-hour light/dark cycles.

### Chemicals

**Figure 1:**
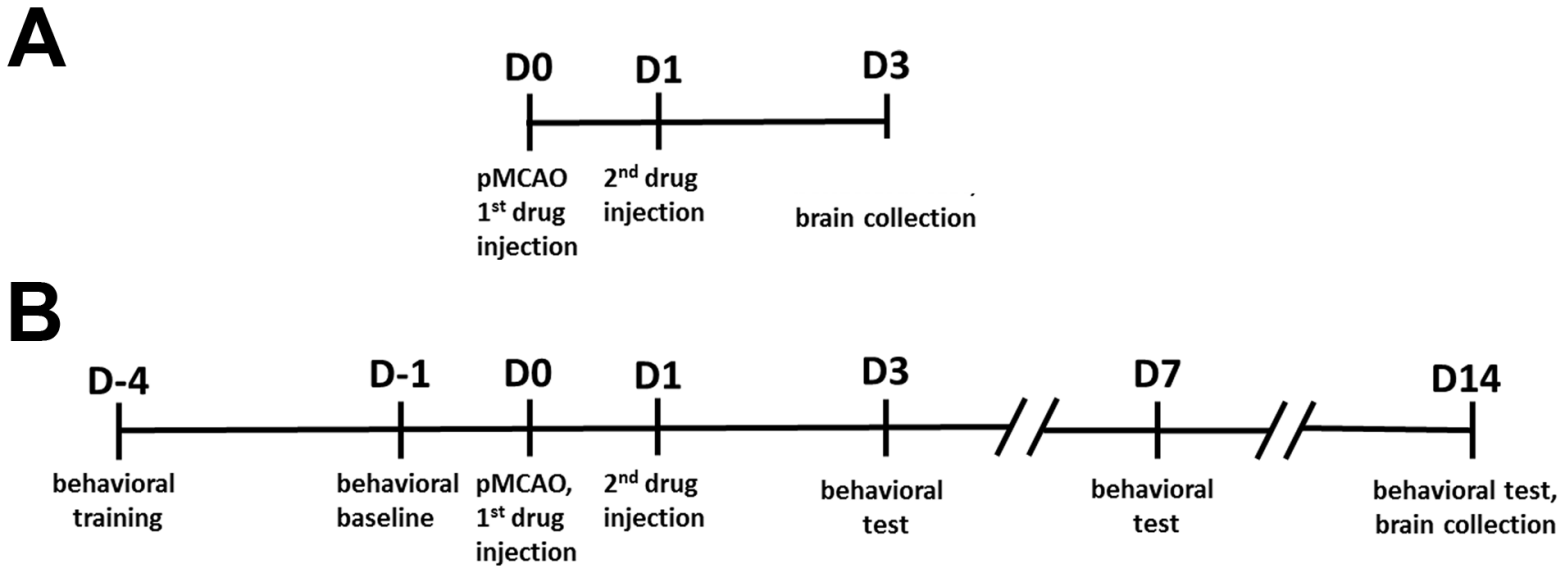
Experimental design. A: Drugs were injected intra-peritoneally immediately (1^st^ injection) and 24 hours (2^nd^ injection) after pMCAO. Brain samples were collected 3 days after pMCAO. B: All mice were trained for behavioral tests 4 days before pMCAO. Behavioral performance at baseline was recorded 1 day before pMCAO (D-1) and behavioral tests were conducted on D3, D7 and D14 after pMCAO. Drugs were injected intra-peritoneally immediately (1^st^ injection) and 24 hours (2^nd^ injection) after pMCAO. Brain samples were collected 14 days after pMCAO.

PHA 568487 (PHA), a selective α-7 nAchR agonist, was purchased from Tocris Bioscience (Ellisville, MO), and methyllycaconitine (MLA), an α-7 nAchR antagonist, from Sigma (St Louis, MO). Both were diluted with 0.9% saline prior to the experiment, then injected intra-peritoneally as shown in Figure 1.

### Permanent occlusion of distal middle cerebral artery (pMCAO)

Mice were anesthetized with 2% isoflurane inhalation. Buprenorphine (0.1 mg/kg of body weight) was injected intraperitoneally. Under a surgical microscope, a 1-cm incision was made between the left orbit and tragus and a 2 mm^2^ craniectomy was performed. The arachnoid mater was opened, and the left middle cerebral artery was permanently occluded using electrical coagulation just proximal to the pyriform branch. The left MCA and its branches proximal to this point maintained blood flow, and only the segment and branches distal to this point were blocked. During the surgical procedure, body temperature was maintained at 37±0.5°C using a thermal blanket, and systemic blood pressure was monitored using a tail-cuff system from Visitech Systems (Apex). Surface cerebral blood flow (sCBF) was monitored throughout the procedure using a laser Doppler flowmeter (Vasamedics Inc, MN). Mice were excluded from the study if sCBF in the ischemic core region was more than 15% of the baseline [8]. Mice were allowed to recover from the anesthesia on a warm blanket. Brain samples were collected 3 and 14 days after pMCAO. A total of 6 mice had to be euthanized because of massive bleeding during the surgery procedure.

### Behavioral tests

#### Corner test

To detect sensorimotor and postural asymmetries, the corner test was used as previously described [26]. Mice were placed between two boards with identical dimensions (30×20×1 cm^2^). When mice approached the corner, both sides of their vibrissae were stimulated. The mice reared forward and upward, then turned back to face the open end. Normal mice would turn to the left or right side with equal frequency, whereas stroke mice would turn more frequently to the lesion side (left side in this study). The percentage of left turns was recorded in 3 different sets of 10 trials 1 day before pMCAO (baseline), and 3, 7 and 14 days after. Turning movements without incorporating a rearing movement were not recorded.

#### Adhesive removal test

As previously described [27], the adhesive removal test was performed to assess possible somatosensory deficits. Adhesive tape (0.3×0.3 cm) was applied on each paw. The time was recorded with a maximum testing time of 120 seconds, after which the tapes were removed from each paw. Mice were trained twice daily for 4 days before the surgery procedure in order to obtain an optimal level of performance. The adhesive time was recorded after 2 rounds of trials performed 1 day before pMCAO (baseline), and 3, 7 and 14 days after.

### Evaluation of infarct and atrophy volume

Lesion volume (infarct volume 3 days and atrophy volume 14 days after pMCAO) was quantified on cresyl violet-stained sections. Briefly, a series of 20- µm thick coronal sections, from bregma 1.7 mm to 2.1 mm (about 8 mm^3^), were obtained. One in every 10 sections was stained with cresyl violet. Images were taken from the brain sections and digitized. Using image J, the infarct and atrophy areas were outlined and their areas, quantified. The infarct volumes were reconstructed by taking the sum of infarct areas from all these sections and multiplying by 200 µm. The atrophic area was calculated as the area of the normal area of the ischemic hemisphere subtracted from the non-ischemic hemisphere. The atrophy volume was reconstructed using serial sections [28].

### Immunohistochemical analysis

Immunohistochemical staining was performed using a series of 20- µm-thick coronal sections. For immunofluorescent staining, sections were incubated overnight at 4°C with the following primary antibodies: CD68 (1∶50, AbD Serotec, Raleigh, NC), NeuN (Neuronal Nuclei, 1∶500, Millipore, Bedford, MA), Iba-1 (1∶200, Wako, Richmond, VA), CD11b (1∶200, AbD Serotec, Raleigh, NC), CD206 (1∶100, R&D, Minneapolis, MN), and phosphor-NF-kB p65 (1∶100, Cell Signaling, Danvers, MA). After washing with phosphate-buffered saline (PBS), sections were then incubated with Alexa Fluor 594-conjugated, or Alexa Fluor 488-conjugated IgG (1∶500, Invitrogen, Carlsbad, CA). Terminal deoxynucleotidyl transferase-mediated dUTPnick end-labeling (TUNEL) assays (Apop Tag, Millipore, Bedford, MA) were used to stain apoptotic nuclei according to the manufacture instruction. Negative controls were performed by omitting the primary or the secondary antibodies during the staining procedure. All sections used for quantification were from the same anatomical region (bregma 1.2 to 1.4 mm). The CD68, CD11b, CD206, NF-kB and Iba-1 positive cells were separately counted using Image J software (NIH, USA) by 3 researchers who had no knowledge of the group assignment.

### Real-time RT-PCR analyses

To prevent blood contamination, mice were perfused with saline for 5 minutes to wash out blood from the cerebral vasculature before sample collection. The cortex from bregma 1.7 mm to −2.1 mm (about 8 mm^3^) was rapidly collected under a dissecting microscope, and placed in RNAlater solution (Qiagen, Valencia, CA). Total RNA was extracted using Trizol Reagent (Qiagen, Valencia, OH), and reverse-transcribed into cDNA using a High Capacity RNA to-cDNA Kit (Applied Biosystems, CA). Real-time PCR was performed using TaqMan Fast Advanced Master Mix (Applied Biosystems, CA). Gene-specific primers and probes purchased from Applied Biosystems were used: GAPDH (Mm99999915_g1), CD11b (Mm00434455_m1), iNOS (Mm00440502_m1), CD206 (Mm00485148_m1), SOD1 (Mm01344233_g1), GPX1 (Mm00656767_g1), gp91phox (Mm01287743_m1), and p22phox (Mm00514478_m1). All the samples were run in triplicate, and relative gene expression was calculated using the comparative threshold cycle (CT) and normalized to GAPDH (ΔCT). Results are exhibited as fold-change relative to the mean of the saline-treated group on day 3 after pMCAO.

### Statistical analysis

Data are presented as mean ± SD. Normality was tested with the d'Agostino-Pearson omnibus normality test. Equalities of variances were tested with the F test.

Sample size was determined based on our previous study [8], wherein the time it took to remove the adhesive on the right paw (contralateral to the brain lesion) on day 3 was used as the primary outcome for behavior tests. We estimated that a sample of 10 C57BL/6J MCAO mice per group would be needed to demonstrate a 20% increase (from 20 seconds to 30 seconds with 7 seconds of standard deviation), with 80% power at the 0.025 alpha level (after adjusting for three comparisons, saline vs. PHA, saline vs. MLA) to find a significant difference. We used 11 mice per group for behavior tests. After the final tests 14 days after pMCAO, 7 were used for histological analysis and 4 for gene expression analysis. An additional group of mice (11 for each treatment) was utilized for sample collections 3 days after pMCAO (Figure 1).

Mice are randomly assigned to experimental groups after the baseline behavior tests performed one day before pMCAO. The behavior tests were performed on days 3, 7 and 14 after pMCAO, and the histological, immunostaining and RT-PCR analyses, on days 3 and 14. We performed two-way analysis of variance (ANOVA) for repeated measures to study the timing (days when the test were performed) and the treatment (saline, MLA and PHA) effects. The ANOVA analysis was followed by 3 (behavior) or 2 (other tests) pairwise t-tests with Bonferroni-correction, alpha = 0.05/2 = 0.025 after two-way ANOVA on days 3, 7 and 14 (behavior) and days 3 and 14 (other tests). Thus, P value <0.025 was considered statistically significant.

## Results

### PHA alleviated the sensorimotor deficit

**Figure 2:**
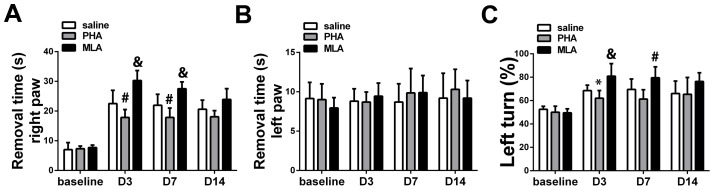
PHA alleviated behavioral dysfunction. A: Adhesive removal tests (right paw). #: p<0.01, &: p<0.001 vs. saline group at the corresponding time points. B: Adhesive removal tests (left paw). C: Corner test. *: p = 0.02, #: p<0.01, &: p<0.001 vs. saline group at the corresponding time points. Baseline recorded 1 day before pMCAO.

Adhesive removal and corner tests were used to determine the influence of PHA (α-7 nAchR agonist) on functional recovery after ischemic injury, as these are the most reliable tests for the pMCAO model [26], [27], [29]. Two-way ANOVA analysis of the right paw removal time (contralateral to the bran lesion), using the adhesive removal test, revealed significant effects on the timing (41.1% of total variation, P<0.0001) and on the treatments (14.7% of total variation, P<0.0001), with significant interaction between the two factors (7.2% of total variation P<0.001). Bonferroni post-hoc analyses showed that, compared to the saline group, PHA mice took significantly less time to remove the adhesive on the right paw on day 3 (18±3 seconds [s] vs. 23±4 s, p = 0.008) and day 7 (18±3 s vs. 22±4 s, p = 0.005) after pMCAO. Although, the PHA group showed reduced adhesive removal time 14 days after pMCAO, the difference between the PHA and saline groups was not statistically significant (18±2 s vs. 21±3 s, p = 0.03, Figure 2A). Conversely, MLA (α-7 nAchR antagonist) mice took longer to remove the adhesive on the right paw on days 3 (31±3 s, p<0.001) and 7 (28±2 s, p<0.001), but not on day 14 (24±4 s, p = 0.03, Figure 2A). Neither treatment had an effect on adhesive removal from the left paws (P>0.05, Figure 2B).

Two-way ANOVA analysis revealed that the timing (68.1% of total variation P<0.0001) and the treatments (13.9% of total variation P<0.0001) also affected the corner test results, with significant interaction between the two factors (5.6% of total variation, P<0.001). Bonferroni post-hoc analyses showed that PHA mice made statistically fewer left turns on day 3 (62±7% vs. 69±5%, p = 0.02). However, the difference did not reach the statistical cut-off on day 7 (61±8% vs. 70±9%, p = 0.03) and was not significant by day 14 (66±11% vs. 65±14%, p = 0.9) after pMCAO (Figure 2C). Conversely, as shown in figure 2C, MLA mice made more left turns than the saline group on day 3 (81±11% p = 0.0003) and day 7 (80±9%, p = 0.003). The difference did not reach the statistical cut-off on day 14 (76±7%, p = 0.028).

### PHA reduced lesion volume and neuronal death

**Figure 3:**
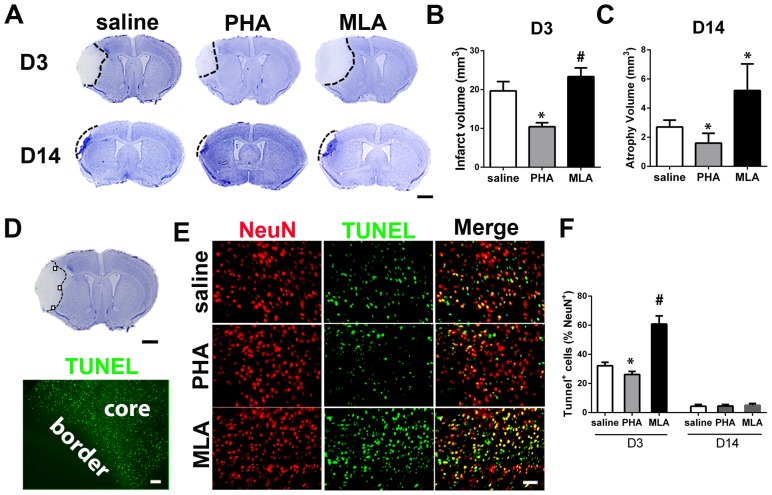
PHA reduced lesion volume and TUNEL^+^ neurons. A: Representative images of cresyl violet-stained sections on D3 and D14 after pMCAO. Scale bar: 1 mm. B: Quantification of infarct volume on D3. *: p = 0.009, #: p = 0.001 vs. saline group. C: Quantification of atrophy volume on D14 after pMCAO. *: p = 0.008 vs. corresponding saline groups. D: A cresyl violet-stained coronal section (bregma 1.3 mm, top, scale bar: 1 mm) and a TUNEL-stained section (bottom, scale bar: 50 um). Black squares in the cresyl violet-stained section show the areas used for quantification of NeuN^+^/TUNEL^+^ cells. Infarct border is shown in the TUNEL-stained section. E: Representative images of NeuN and TUNEL-stained sections. F: Quantification of NeuN and TUNEL double positive cells. *: p = 0.001, #: p<0.001 vs. saline group.

To analyze neuronal injury, infarct and atrophic volume were quantified using cresyl violet-stained brain sections collected 3 (infarct) and 14 (atrophic) days after pMCAO. PHA mice had smaller infarct volume on day 3 (10.2±3.4 mm^3^ vs. 16.0±3.7 mm^3^, p = 0.009, Figure 3A & B) and atrophy volume on day 14 (1.6±0.7 mm^3^ vs. 2.7±0.5 mm^3^, p = 0.008, Figure 3A & C), compared with the saline group at the corresponding time points, whereas MLA mice had larger infarct volume on day 3 (23.3±2.3 mm^3^, p = 0.001, Figure 3A & B) and atrophic volume on day 14 (5.2±1.8 mm^3^, p = 0.008, Figure 3A & C) than the corresponding saline groups. PHA also reduced the number of TUNEL^+^ neurons in the peri-infarct region (Figure 3D) of brain sections collected 3 days after pMCAO (26±2% of NeuN^+^ cells vs. 32±2%, p = 0.001, Figure 3E & F). MLA mice had more TUNEL^+^ neurons (61±6%, p<0.001, Figure 3E & F). All groups had a similar number of TUNEL^+^ neurons on day 14 (PHA group: 5±1% vs.4±1%, p = 0.80; MLA group: 5±2%, p = 0.37, Figure 3F).

### PHA decreased CD68^+^ cells in the peri-infarct region

**Figure 4:**
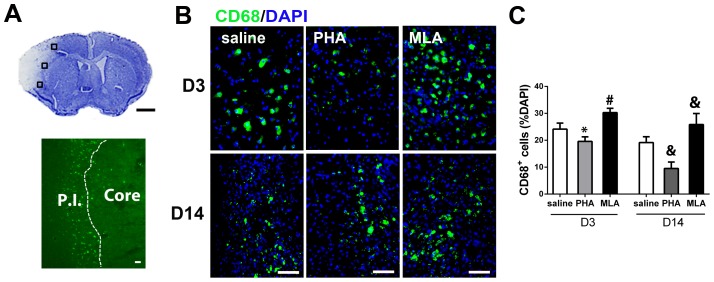
PHA decreased CD68^+^ cells in the peri-infarct region. A: An image of cresyl violet-stained coronal section shows the areas used for cell-quantification (top, squared regions, scale bar: 1 mm) and an image of CD68 antibody-stained section shows the infarct border (bottom, white dotted line, scale bar: 50 um). P.I.: peri-infarct region. B: Representative images show the CD68^+^ cells in the peri-infarct region. The nuclei were counterstained with 4',6-diamidino-2-phenylindole (DAPI). C: Bar graph shows the percentage of CD68^+^ cells among total cells (DAPI positive nuclei) in the peri-infarct region. *: p = 0.001, #: p<0.001 vs. saline group 3 days after pMCAO; &: p<0.001 vs. saline group 14 days after pMCAO.

The total number of CD68^+^ cells in the peri-infarct area was quantified in the area shown in Figure 4A. We found that PHA mice had fewer CD68^+^ cells in the peri-infarct area compared with mice in the saline group both on days 3 and 14 (D3: 20±2% vs. 24±2% of total cells, p = 0.001; D14: 9±3% vs. 16±2%, p<0.001, Figure 4B & C) after pMCAO. The number of CD68^+^ cells significantly increased in the MLA group compared with the saline group on both day 3 and day 14 (D3: 30±2% of total cells, p<0.001; D14: 26±4%, p<0.001, Figure 4B & C) after pMCAO.

### PHA decreased M1 microglia/macrophages and M1/M2 ratio

**Figure 5:**
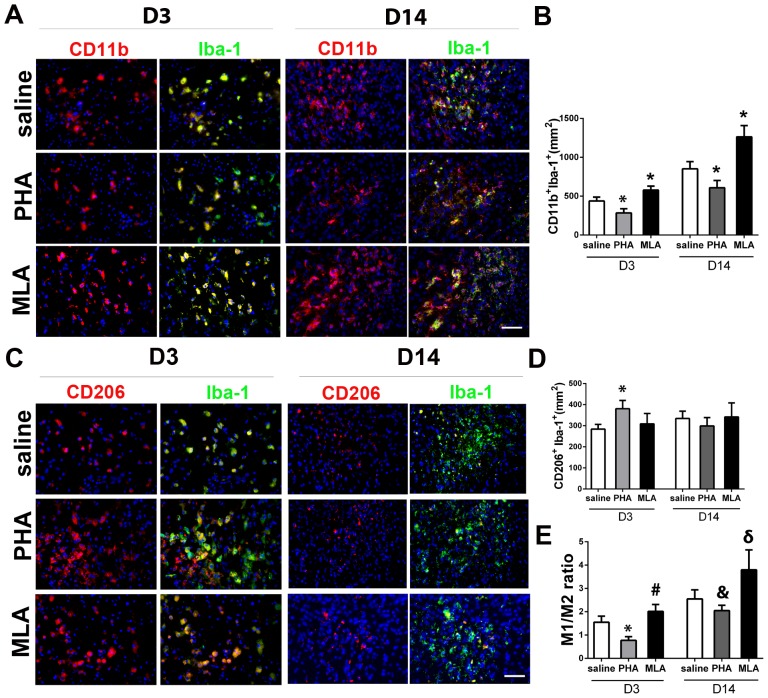
PHA decreased pro-inflammatory microglia/macrophages (M1). A: Representative images of M1 (CD11b^+^Iba-1^+^) staining. The nuclei were counterstained with DAPI. Scale bar: 50 µm. B: Quantification of M1 in the peri-infarct region. *: p<0.001, vs. saline group at corresponding time points. C: Representative images of M2 (CD206^+^Iba-1^+^) staining. The nuclei were counterstained with DAPI. Scale bar: 50 µm. D: Quantification of M2 microglia/macrophages in the peri-infarct region. *: p<0.001 vs. saline group on D3 after pMCAO. E: The ratios of M1 and M2 cells. *: p<0.001, #: p = 0.018 vs. saline group 3 days after pMCAO; and &: p = 0.015, δ: p = 0.009 vs. saline group 14 days after pMCAO.

To analyze whether PHA treatment alters microglia/macrophage polarization, we used antibodies specific to CD11b and Iba-1 to identify M1, and antibodies specific to CD206 and Iba-1 to identify M2. Compared with the saline group, PHA mice had fewer M1 3 days (284±54/mm^2^ vs. 437±50/mm^2^, p<0.001) and 14 days (609±94/mm^2^ vs. 852±92/mm^2^, p<0.001, Figure 5A & B) after pMCAO. The MLA group had more M1 than the saline group 3 days (578±52/mm^2^, p<0.001) and 14 days (1263±145/mm^2^, p<0.001, Figure 5A & B) after pMCAO. PHA significantly increased the M2 cells on day 3 (381±39/mm^2^ vs. 284±23/mm^2^, p<0.001, Figure 5C & D) compared with the saline group, whereas MLA did not alter the number of M2. M2 numbers were similar in all groups 14 days after pMCAO (PHA: 299±40/mm^2^ vs. 335±34/mm^2^, p = 0.12; MLA: 342±66/mm^2^, p = 0.81, Figure 5D). Thus, M1/M2 ratios were lower in the PHA group on day 3 (0.8±0.3 vs. 1.6±0.3, p<0.001) and day 14 (2.0±0.8 vs. 2.6±1.0, p = 0.015), and higher in the MLA group on day 3 (2.0±0.3, p = 0.018) and day 14 (3.8±1.6, p = 0.009), compared with the saline groups (Figure 5E).

### PHA decreased M1 and increased M2 marker gene expression

**Figure 6:**
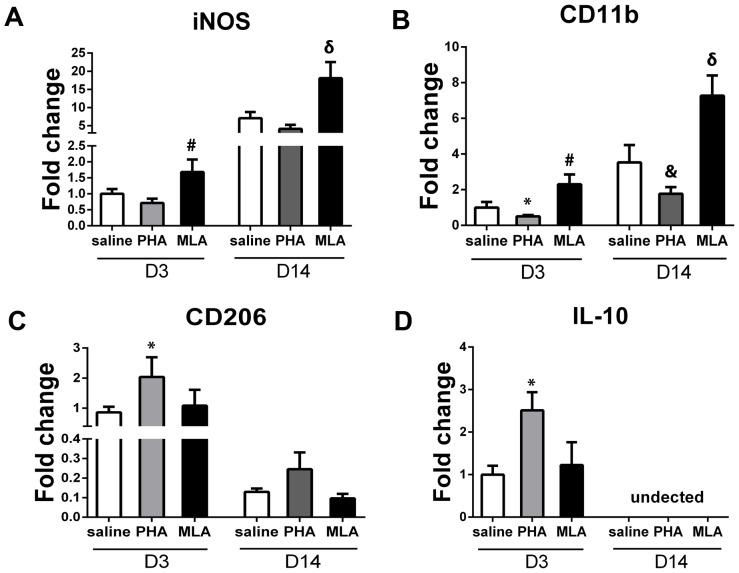
PHA decreased M1 marker expression and increased M2 marker expression. A: Quantification of iNOS. #: p = 0.017 vs. saline group 3 days after pMCAO; and δ: p = 0.004 vs. saline group 14 days after pMCAO. B: Quantification of CD11b. *: p = 0.021, #: p = 0.006 vs. saline group 3 days; and &: p = 0.003, δ: p = 0.002 vs. saline group 14 days after pMCAO. C: Quantification of CD206. *: p = 0.01 vs. saline group 3 days after pMCAO. D: Quantification of IL-10. *: p<0.001 vs. saline group on D3 after pMCAO.

To corroborate the histological findings, we quantified the expression of M1 markers (iNOS and CD11b) and M2 markers (CD206 and IL-10) using real time RT-PCR in the tissues collected from the peri-infarct area. PHA mice expressed lower iNOS but did not reach the Boferroni post-hoc cut-off adjusted for multiple comparison (day 3: 0.7±0.1-fold vs. 1.0±0.1-fold, p = 0.03; day 14: 4.1±1.1-fold vs. 7.0±1.7-fold, p = 0.03, Figure 6A). However, PHA significantly decreased CD11b expression (day 3: 0.5±0.1-fold vs. 1.0±0.3-fold, p = 0.021; day 14: 1.8±0.4-fold vs. 3.5±1.0-fold, p = 0.003, Figure 6B). Regarding M2 markers, PHA increased CD206 (2.0±0.7-fold vs. 0.9±0.2-fold, p = 0.01, Figure 6C) and IL-10 (2.5±0.4-fold vs. 1.0±0.2-fold, p<0.001, Figure 6D) expression on day 3 compared with corresponding saline groups. However, the increased expression of CD206 in the PHA group did not reach the statistical cut-off 14 days after pMCAO (0.2±0.1-fold vs. 0.1±0.02-fold, p = 0.03, Figure 6C). We could not detect IL-10 expression in the samples collected 14 days after pMCAO. These results were consistent with the histological analysis. Conversely, the MLA group expressed higher iNOS (day 3: 1.7±0.4-fold, p = 0.017; day 14: 18.1±4.4-fold, p = 0.004, Figure 6A) and CD11b (day 3: 2.3±0.6-fold, p = 0.006; day 14: 7.3±1.1-fold, p = 0.002, Figure 6B). MLA treatment did not change CD206 expression 3 and 14 days (p>0.05, Figure 6C) and IL-10 expression 3 days (P = 0.46, Figure 6D) after pMCAO.

### PHA increased anti-oxidant gene expression and decreased NADPH oxidase and NF-kB activity

**Figure 7:**
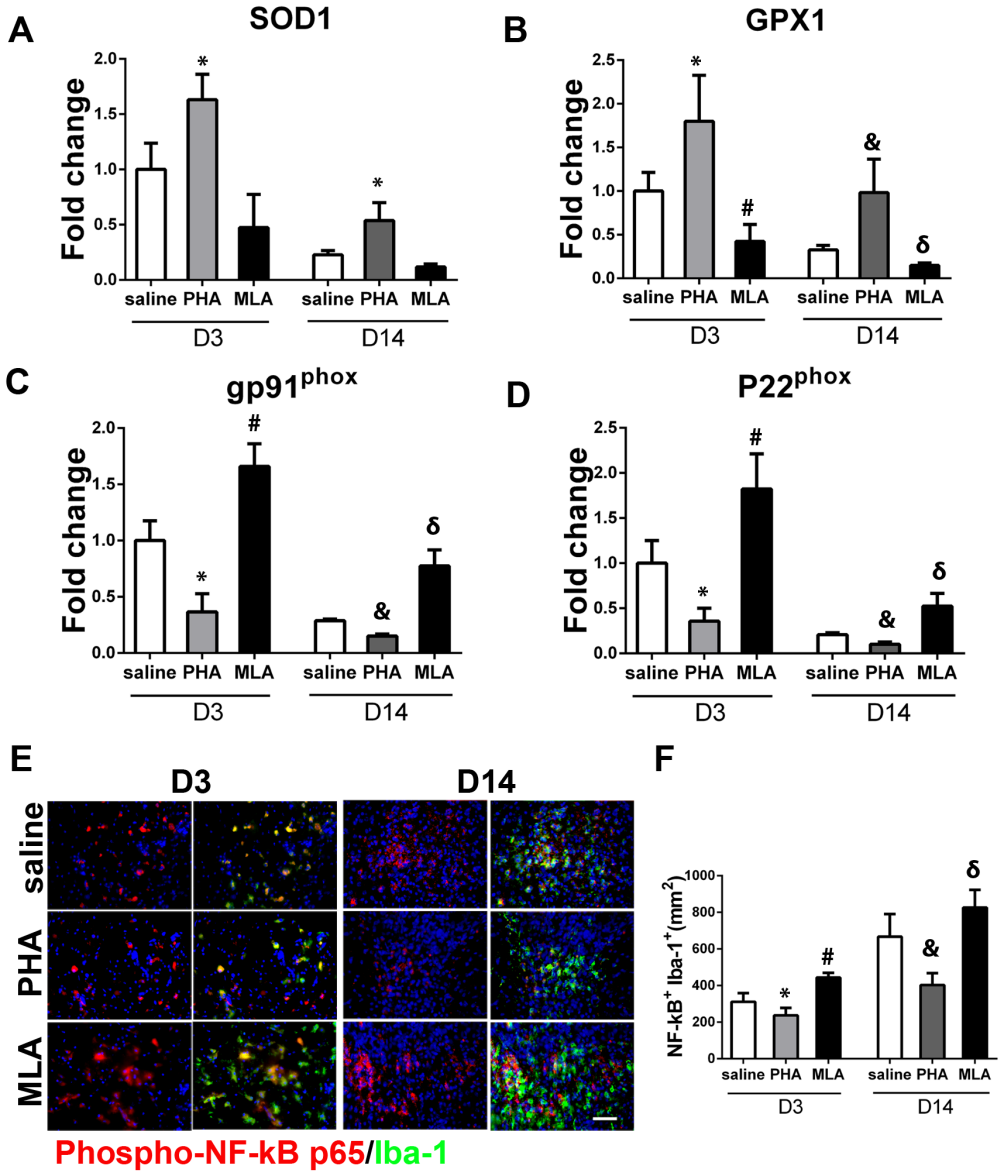
PHA increased anti-oxidant gene expression and decreased NADPH oxidase and phospho-NF-kB p65^+^ microglia/macrophages. A: Quantification of SOD1. *: p = 0.009 vs. corresponding saline groups. B: Quantification of GPX1. *: p = 0.013, #: p = 0.007 vs. saline group on D3; and &: p = 0.01, δ: p<0.001 vs. saline group on D14 after pMCAO. C: Quantification of gp91^phox^. *: p = 0.002, #: p = 0.003 vs. saline group on D3; and &: p<0.001, δ: p<0.001 vs. saline group on D14 after pMCAO. D: Quantification of p22^phox^. *: p = 0.004, #: p = 0.01 vs. saline group on D3; and &: p<0.001, δ: p = 0.005 vs. saline group on D14 after pMCAO. E: Representative images show phospho-NF-kB p65^+^Iba-1^+^ cells in the peri-infarct region on D3 and D14 after pMCAO. Scale bar: 50 um. F: Quantification of phospho-NF-kB p65^+^Iba-1^+^ cells. *: p = 0.01, #: p<0.001 vs. saline group on D3; and &: p<0.001, δ: p = 0.019 vs. saline group on D14 after pMCAO.

The expression of anti-oxidant genes (superoxide dismutase 1 [SOD1] and glutathione peroxidase 1 [GPX1]), and the expression of the two subunits of pro-oxidative stress protein NADPH oxidase (gp91phox and p22phox) were analyzed using RT-PCR. NF-kB activity was analyzed by quantifying phospho-NF-kB p65 positive microglia/macrophages in the peri-infarct region. PHA increased the expression of SOD1 (day 3: 1.6±0.2-fold vs. 1.0±0.2-fold, p = 0.009; day 14: 0.5±0.2-fold vs. 0.2±0.04-fold, p = 0.009, Figure 7A) and GPX1 (day 3: 1.8±0.5-fold vs. 1.0±0.2-fold, p = 0.013; day 14: 1.0±0.4-fold vs. 0.3±0.1-fold, p = 0.01, Figure 6B), and decreased the expression of gp91^phox^ (day 3: 0.4±0.2-fold vs. 1.0±0.2-fold, p = 0.002; day 14: 0.2±0.02-fold vs. 0.3±0.01-fold, p<0.001, Figure 6C) and p22^phox^ (day 3: 0.4±0.1-fold vs. 1.0±0.3-fold, p = 0.004; day 14: 0.1±0.03-fold vs. 0.2±0.02-fold, p<0.001, Figure 6D), compared with the saline group at the corresponding time points. MLA did not significantly decrease SOD1 expression (day 3: 0.5±0.3-fold, p = 0.03; day 14: 0.1±0.03-fold, p = 0.04, Figure 6A). However, the decrease in GPX1 expression was significant (day 3: 0.4±0.2-fold, p = 0.007; day 14: 0.1±0.03-fold, p<0.001, Figure 6B), as well as the increase in the expression of gp91^phox^ (day 3: 1.7±0.2-fold, p = 0.003; day 14: 0.8±0.1-fold, p<0.001, Figure 6C) and p22^phox^ (day 3: 1.8±0.4-fold, p = 0.01; day 14: 0.5±0.1-fold, p = 0.005, Figure 6D), compared with the saline group at the corresponding time points. PHA also decreased phospho-NF-κB p65^+^ microglia/macrophages (Iba 1^+^) on day 3 (237±44/mm^2^ vs. 311±52/mm^2^, p = 0.01) and day 14 (403±65/mm^2^ vs. 667±123/mm^2^, p<0.001, Figure 7E & F) in the peri-infarct region compared to corresponding saline groups, whereas MLA treatment increased phospho-NF-κB p65^+^ microglia/macrophages on day 3 (443±28/mm^2^, p<0.001) and day 14 (827±96/mm^2^, p = 0.019, Figure 7E & F).

## Discussion

In our study, we found that in mice, activation of α-7 nAchR by PHA at the acute stage of ischemic stroke reduced brain injury and functional deficits after pMCAO. PHA treatment also reduced the number of CD68^+^, pro-inflammation M1 microglia/macrophages (CD11b^+^/Iba-1^+^), and M1 marker genes (CD11b, iNOS) in the peri-infarct region. As a result, the ratio of pro-inflammation M1 to anti-inflammation/pro-tissue repair M2 was reduced in the PHA group. Further, PHA increased anti-oxidant gene expression and decreased expression of NADPH oxidase, which is associated with reduced expression of NF-kB activity in microglia/macrophages in the peri-infarct region. Our data therefore suggest that reduction of pro-inflammation microglia/macrophages and oxidative stress are part of the underlying mechanisms of α-7 nAchR neuro-protective effect.

Inflammation has biological roles in determining the outcome of stroke. Both systemic and local inflammation in the acute phase of ischemic stroke may have deleterious effects on stroke outcome [3]–[5]. Prolonged systemic inflammation increases functional impairment after focal ischemia in rats [30]. Therefore, reducing inflammation poses a therapeutic opportunity for improving functional outcomes in stroke patients.

Since most of the commonly used anti-inflammatory drugs (including non-steroidal [NSAID] and steroids) are associated with some side effects (e.g., increasing the risk of stroke) and are not suitable for use in stroke patients [31]–[33], it is necessary to explore new strategies. Recently, the cholinergic pathway has been found to inhibit cytokine release through a mechanism requiring nAChRs [34]. Activation of nAChR protects against ischemic stroke-related cerebral damage [35], [36] and reduces tibial fracture-induced systemic/hippocampal inflammation [21]. Activation of α7 nAchR has also been shown to reduce neuronal death in a subarachnoid hemorrhage rat model [16] as well as in an intracerebral hemorrhage mouse model [19]. We demonstrated in this study that activation of α-7 nAchR using PHA reduces lesion volume and apoptotic neuron, and improves functional recovery in ischemic stroke mice. Both microglia and peripheral macrophages express α7 nAchR [15], [37]. Since we delivered PHA via intra-peritoneal injection, we postulate that the effect we observed in this study is mostly mediated by systemic macrophage α-7 nAchR.

Macrophages participate in acute inflammatory response to infection and tissue injuries, and give rise to different cell populations that participate in host defense (M1, pro-inflammation), wound healing (M2, anti-inflammation), and immune regulation [38]. Deletion of the macrophage lineage may cause deleterious effects on stroke recovery. Exaggerated host defense, such as an excessive amount of M1 macrophages in the ischemic brain injury area at the recovery stage, may have an adverse impact on injury repair. Thus, modulating host response by reducing M1 and increasing M2 at the recovery stage could play a role in α-7 nAchR neuro-protective effect. In this study, we demonstrated that activation of α-7 nAchR using PHA at the acute stage not only reduced CD68^+^ and M1 cells in the peri-infact region at both the early and late stages of ischemic stroke, but also increased M2 cells at the early stage, resulting in a decrease in the M1/M2 macrophage ratio. With the α-7 nAchR antagonist (MLA), however, both M1 macrophages and the M1/M2 ratio increased, and no effect was observed in M2 macrophages.

NADPH oxidase (NOX)-mediated oxidative stress has been shown to play a major role in pathogenic actions following ischemic stroke [22], [23] by contributing to the inflammatory response. NOX2-deficient mice have lower levels of pro-inflammatory mediators than wild-type mice [24]. Also, the inflammatory transcription factor, NF-kB, participates in the oxide stress process [25]; however, the connection between α-7 nAchR and oxide stress has not been demonstrated. Our data show that activation of α-7 nAchR reduces oxidative stress and NF-kB activity.

The limitations of this study are: (1) The first dose of PHA was administered immediately after pMCAO, which is almost impossible to perform in a clinical setting. More clinically relevant treatment schemes, such as giving the first dose between 6–24 hours after stroke, will be tested in future studies. (2) We used 11 mice per group for behavioral analyses based on our previous data. With this sample size, the PHA group showed better performance at 14 days for right paw adhesive removal (P = 0.03), and 7 days for the corner test (P = 0.03). However, the P values of these tests at the indicated time-points did not reach the significant cutoff point we have set (P = 0.025). (3) It was unclear why MLA had no effect on M2 macrophage numbers. One possible explanation is that different doses may be needed to induce different phenotypes in various models. For example, 6 mg/kg of MLA exacerbated the behavioral dysfunction in our model, but did not increase behavioral dysfunction or brain edema in a mouse model of intracerebral hemorrhage in another study [19]. A greater dose of MLA might be needed to reduce the number of M2 microphages.

In conclusion, our study has determined that reduction of pro-inflammatory M1, oxidative stress, and NF-kB activity in microglia/macrophages contributes to the neuro-protective effect of α-7 nAchR. Moreover, the data from this study posit a strong case for considering α-7 nAchR activation a potential and viable therapeutic opportunity to improve functional recovery in ischemic stroke patients.
