# Correction: Activation of α-7 Nicotinic Acetylcholine Receptor Reduces Ischemic Stroke Injury through Reduction of Pro-Inflammatory Macrophages and Oxidative Stress

**DOI:** 10.1371/journal.pone.0152218

**Published:** 2016-03-17

**Authors:** Zhenying Han, Fanxia Shen, Yue He, Vincent Degos, Marine Camus, Mervyn Maze, William L. Young, Hua Su

In preparing this article for publication, the CD206-Saline panel in Fig 5C and the NeuN-Saline panel in Fig 3E were mistakenly duplicated from another of the authors’ publications that was prepared at the same time:

Han Z, Li L, Wang L, Degos V, Maze M, Hua S (2014) Alpha-7 nicotinic acetylcholine receptor agonist treatment reduces neuroinflammation, oxidative stress, and brain injury in mice with ischemic stroke and bone fracture. Journal of Neurochemistry 131: 498–508. doi: 10.1111/jnc.12817

The authors have provided corrected versions of Figs 3 and 5 here, both of which include the correct images for the aforementioned panels. The raw images used to create the corrected panels and additional data can be viewed on the Harvard Dataverse (https://dataverse.harvard.edu/dataverse/Han). The authors confirm that this error does not alter their results.

**Fig 3:**
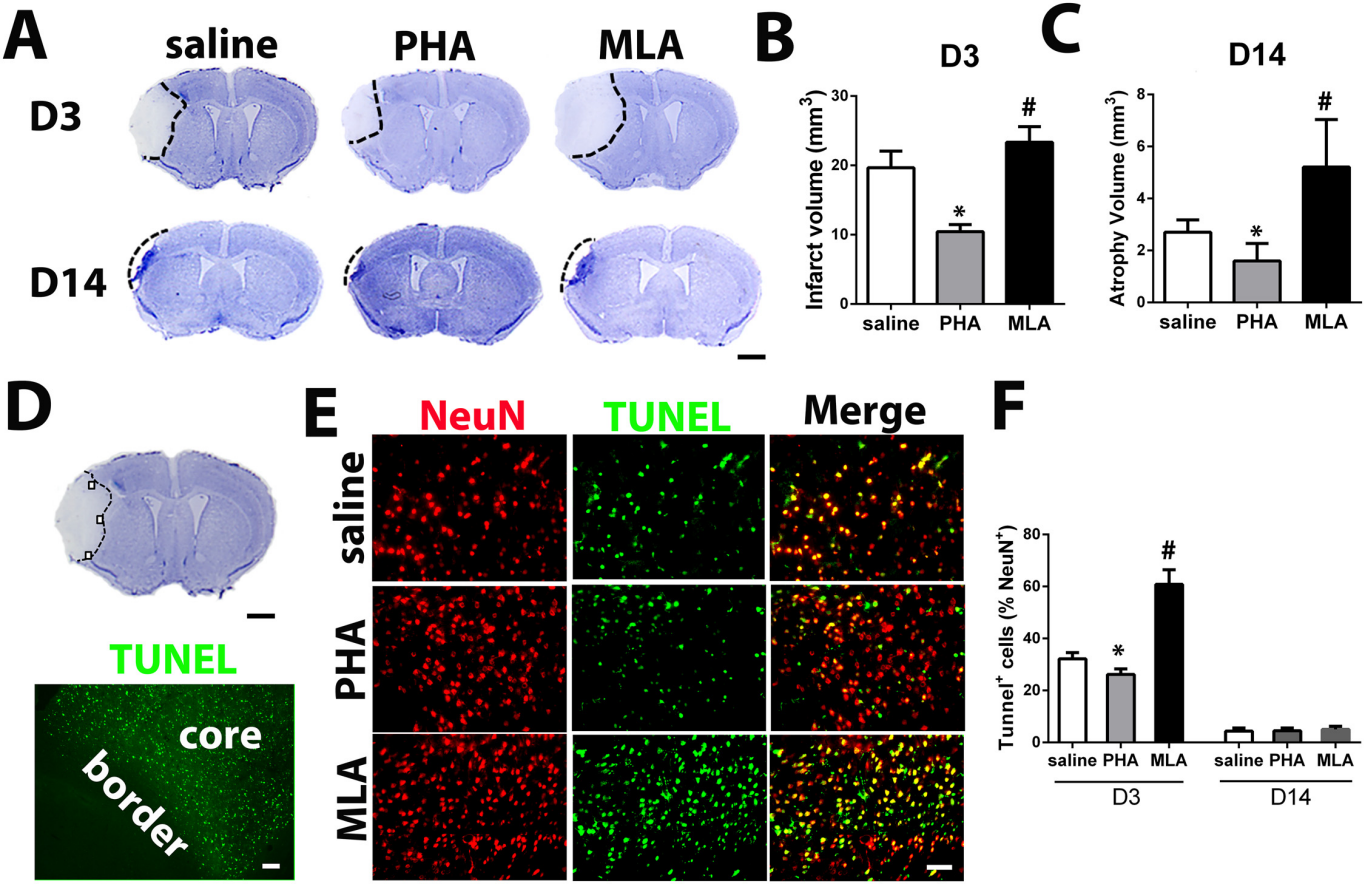
PHA reduced lesion volume and TUNEL^+^ neurons. A: Representative images of cresyl violet-stained sections on D3 and D14 after pMCAO. Scale bar: 1 mm. B: Quantification of infarct volume on D3. *: p = 0.009, #: p = 0.001 vs. saline group. C: Quantification of atrophy volume on D14 after pMCAO. *: p = 0.008 vs. corresponding saline groups. D: A cresyl violet-stained coronal section (bregma 1.3 mm, top, scale bar: 1 mm) and a TUNEL-stained section (bottom, scale bar: 50 um). Black squares in the cresyl violet-stained section show the areas used for quantification of NeuN^+^/TUNEL^+^ cells. Infarct border is shown in the TUNEL-stained section. E: Representative images of NeuN and TUNEL-stained sections. F: Quantification of NeuN and TUNEL double positive cells. *: p = 0.001, #: p<0.001 vs. saline group.

**Fig 5:**
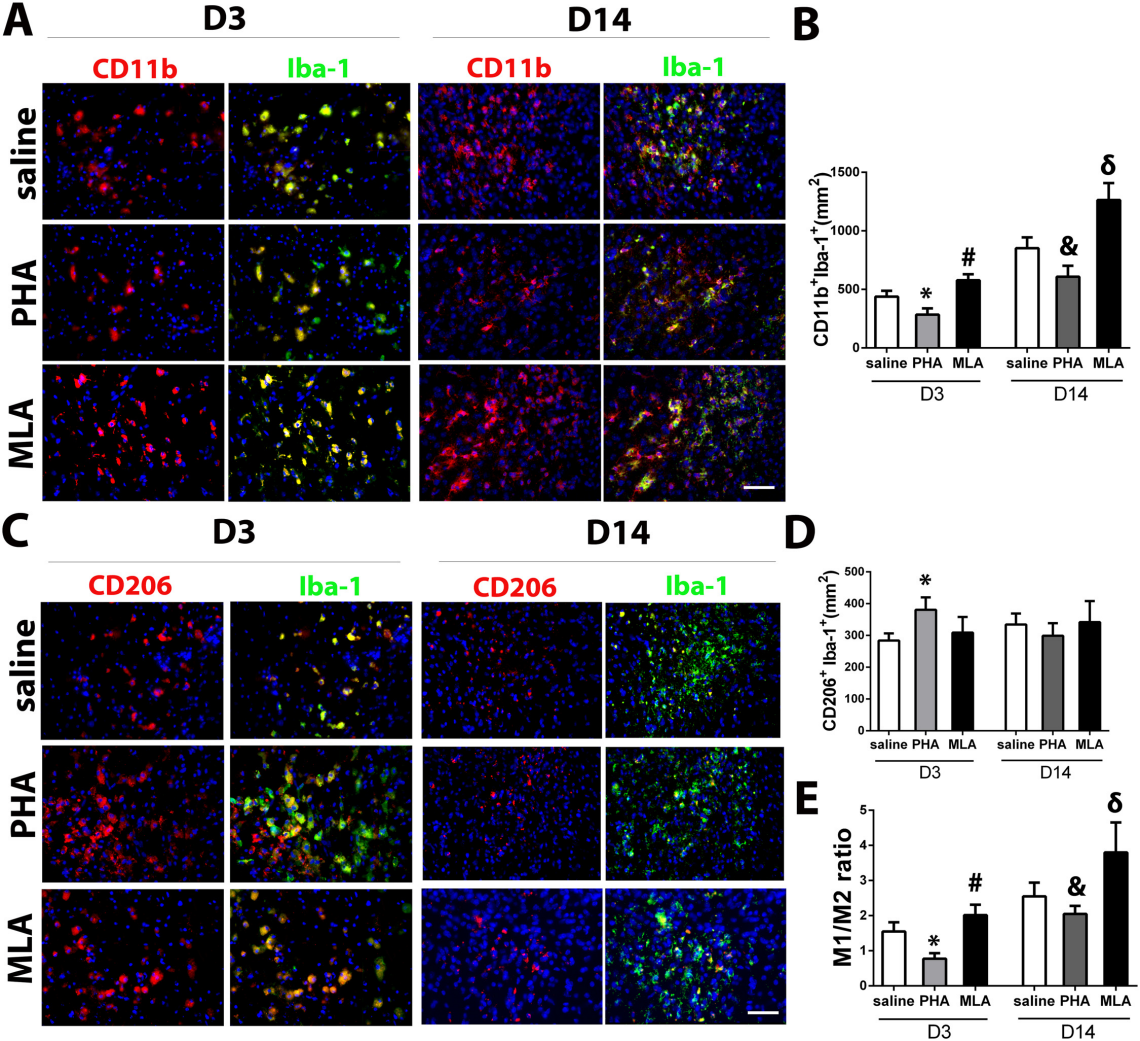
PHA decreased pro-inflammatory microglia/macrophages (M1). A: Representative images of M1 (CD11b^+^Iba-1^+^) staining. The nuclei were counterstained with DAPI. Scale bar: 50 μm. B: Quantification of M1 in the peri-infarct region. *: p<0.001, vs. saline group at corresponding time points. C: Representative images of M2 (CD206^+^Iba-1^+^) staining. The nuclei were counterstained with DAPI. Scale bar: 50 μm. D: Quantification of M2 microglia/macrophages in the peri-infarct region. *: p<0.001 vs. saline group on D3 after pMCAO. E: The ratios of M1 and M2 cells. *: p<0.001, #: p = 0.018 vs. saline group 3 days after pMCAO; and &: p = 0.015, δ: p = 0.009 vs. saline group 14 days after pMCAO.

## References

[1] Han Z, Shen F, He Y, Degos V, Camus M, Maze M, et al (2014) Activation of α-7 Nicotinic Acetylcholine Receptor Reduces Ischemic Stroke Injury through Reduction of Pro-Inflammatory Macrophages and Oxidative Stress. PLoS ONE 9(8): e105711 doi: 10.1371/journal.pone.0105711 25157794 10.1371/journal.pone.0105711 PMC4144901

